# Individual patient data meta-analysis : Cervical stitch (cerclage) for preventing pregnancy loss in women

**DOI:** 10.1186/1471-2393-5-5

**Published:** 2005-02-23

**Authors:** Catrin Tudur-Smith, Andrea L Jorgensen, Zarko Alfirevic, Paula R Williamson

**Affiliations:** 1 Centre for Medical Statistics and Health Evaluation, University of Liverpool, Liverpool, L69 3BX, UK; 2 University Department of Obstetrics and Gynaecology, Liverpool Women's Hospital, Liverpool, UK

## Abstract

**Background:**

Cervical cerclage is a surgical procedure involving suturing the cervix with a purse type stitch to keep it closed during pregnancy. This procedure has been used widely in the management of pregnancies considered at high risk of preterm delivery. Several observational studies into the efficacy of cervical cerclage have claimed high rates of successful pregnancy outcome in women with a poor obstetric history attributed to cervical incompetence. However, a recent aggregate data Cochrane review found no such conclusive evidence from seven included randomised studies. Current data suggests that cervical cerclage is likely to benefit women considered to be 'at very high risk' of a second trimester miscarriage due to a cervical factor, however identifying such women remains elusive and many women may be treated unnecessarily. Undertaking an individual patient data (IPD) meta-analysis of the studies will allow us to investigate whether treatment is more effective in particular subgroups. Such an analysis will also provide a more powerful analysis of the predictors of preterm delivery and pregnancy loss, including ultrasound measurement of cervical length, and will allow a more complete analysis of 'time to event' outcomes.

**Methods/Design:**

The analysis will include data from randomised trials comparing the intervention of elective cerclage versus no cerclage or bedrest to prevent miscarriage or pre-term labour. A specific list of data will be requested for each trial, including demographic and obstetric history data. The primary outcomes of interest will be neonatal mortality/morbidity. Attention will also be given to secondary outcomes such as time from randomisation to delivery, preterm delivery before 32 weeks and maternal morbidity. An intention to treat analysis will be performed, with attention paid to assessing clinical and statistical heterogeneity. Multilevel models with patients and trials as the two levels will be explored to investigate treatment effect on various outcomes. Patient-level covariates will be incorporated into the models in an attempt to account for statistical heterogeneity as well as to investigate interactions with treatment effect.

**Discussion:**

Predictive models generated from our analysis should lead to more effective counselling of women at risk and a more cost effective use of cerclage.

## Background

Cervical cerclage is a surgical procedure carried out during pregnancy. The operation involves suturing the neck of the womb (cervix) with a purse type stitch to keep the cervix closed. This surgical procedure has been used widely in the management of pregnancies considered to be at high risk of preterm delivery.

Several observational studies in the last 50 years have claimed high rates of successful pregnancy outcome in women that had a poor obstetric history attributed to cervical incompetence. However, a recent Cochrane review found no conclusive evidence from seven included randomised studies that inserting a cervical stitch in women perceived to be at risk of preterm birth or second trimester pregnancy loss attributed to cervical factors, reduces the risk of pregnancy loss, preterm delivery or morbidity associated with preterm delivery (Drakeley 2003)[1].

In the Cochrane review, the data for important clinical outcomes including preterm delivery and maternal infection showed significant heterogeneity due to inconsistency in clinical definitions used, including the cut off gestational age defining preterm delivery, and different patient populations studied.

Practically, methods of undertaking a meta-analysis of several studies may involve collecting either aggregate data, or data on each patient individually. The advantages of the latter approach, described as the 'yardstick' (Chalmers 1993)[2] include (i) a more complete analysis of 'time of event' outcomes and (ii) a more powerful analysis of whether treatment is more or less effective in particular subgroups (Stewart 1993)[3].

One of the main concerns regarding current evidence related to cervical cerclage and other interventions for preventions of preterm delivery is a possibility that the 'primary outcomes' may have been selected to give results in greatest accord with the a priori beliefs of the authors. The evidence to support this phenomenon of within-study selective reporting comes from empirical research, which demonstrates discrepancies between research protocols and subsequent publications (Hahn 2002 [4], Williamson 2005 [5], Chan 2004 [6]). Individual patient data (IPD) meta-analysis has the capacity to overcome these problems.

Currently available data suggest that cervical cerclage is likely to be of benefit for women considered 'at very high risk' of second trimester miscarriage due to a cervical factor e.g. greater than two second trimester losses or progressive shortening of the cervix on ultrasound. However, predicting those women who will miscarry due to a cervical factor remains elusive and many women may be treated unnecessarily. The use of IPD will allow us to investigate predictors of preterm delivery including ultrasound measurement of cervical length and other woman-cerclage interactions.

IPD meta-analysis will allow an investigation of the hypothesis that the effect of cerclage is greater on extreme preterm delivery. In addition, an IPD meta-analysis has greater power than a single trial for examining subgroups. The efficacy of a treatment may depend on several factors. For aggregate data, a meta-analysis stratifying by the absolute risk in the control group may be the only method possible for accounting for these multiple factors simultaneously. This analysis is 'flawed and produces seriously misleading results' (Sharp 1996)[7]. A regression analysis of IPD allows the relation between treatment effect and risk score, derived from these multiple risk factors, to be investigated thereby avoiding these problems.

## Methods/Design

### Objectives

The aim of this project is to undertake an IPD meta-analysis of randomised trials of cervical cerclage. Specific objectives are as follows.

1. To estimate the effect of cervical cerclage on gestational age at delivery.

2. To investigate whether cervical cerclage is more likely to prevent extreme prematurity (<28 weeks) or delivery at later gestations.

3. To investigate risk factors for preterm delivery.

4. To investigate interactions between risk factors and cervical cerclage.

5. To model the effect of cervical cerclage and other risk factors on neonatal and maternal morbidity.

### Criteria for considering studies for this IPD meta-analysis

The types of studies considered for inclusion in the analysis will be all randomised trials comparing cervical cerclage with expectant management or no cerclage during pregnancy. The previous Cochrane review (Drakeley 2003) [1] identified eight eligible trials with 2,513 randomised women (Rust 2000 [8], Althuisius 2000 [9], Althuisius 2001 [10], Rush 1983 [11], Lazar 1984 [12], Dor 1982 [13], MRC/RCOG 1988 [14], Meekai To et al. [15]). We have agreement in principle to provide IPD from six of these trials (Meekai To et al. [15], MRC/RCOG 1988 [14], Rust 2000 [8], Althuisius 2000 [9], Althuisius 2001 [10], Rush 1983 [11]), accounting for 1919(78%) of all women randomised. The remaining trialists and trialists of any further trials identified as eligible will be approached at the start of the project and we anticipate their willingness to collaborate. Already, two further trials have been identified (Berghella 2004 [16], Ezechi 2003 [17]) and the authors have agreed to provide IPD from these trials.

The data collected in the studies will relate to women with confirmed, or suspected of having, cervical incompetence who desire future pregnancies and women who present as an emergency and are thought to have a diagnosis of cervical incompetence. The intervention investigated in the studies will be elective cerclage by whichever method (Shirodkar technique, McDonald technique, transabdominal and transvaginal methods), versus no cerclage or bed rest as interventions to prevent miscarriage or pre-term labour as defined in the original Cochrane review (Drakeley 2003)[1].

### Search strategy for identification of studies

The methods of trial identification described in the original Cochrane review (Drakeley 2003)[1] (see below) will be adopted and updated to December 2004.

The original review has drawn on the search strategy developed for the Pregnancy and Childbirth Group. The full list of journals and conference proceedings as well as the search strategies for the electronic databases, which are searched by the Group on behalf of its reviewers, are described in detail in the 'Search strategies for the identification of studies section' within the editorial information about the Cochrane Pregnancy and Childbirth Group. Briefly, the Trials Search Coordinator searches on a regular basis MEDLINE, the Cochrane Controlled Trials Register and reviews the Contents tables of a further 38 relevant journals received via ZETOC, an electronic current awareness service.

In addition, handsearches will be performed on congress proceedings of the International and European society meetings of feto-maternal medicine, recurrent miscarriage and reproductive medicine. Whenever possible, investigators will be contacted to ask about any additional studies potentially eligible for inclusion.

### Trial eligibility and methodological quality assessment

Two reviewers will independently assess eligibility of identified randomised controlled trials for inclusion in the review. Any difference of opinion will be resolved by discussion. The methodological quality of each trial will be assessed by summarising the method of generation of randomisation list, method of allocation concealment, and potential impact of losses to follow-up. Quasi-randomised studies in which allocation was transparent (e.g. use of alternative allocation or medical record numbers) were excluded in the original review.

### Data collection

The following data for each woman/infant pair will be requested from all trials: date of randomisation and gestational age, maternal demographics and obstetric characteristics at randomisation including cervical length on ultrasound, fibronectin and bacterial vaginosis data if available, treatment allocated, complications during pregnancy including ruptured membranes, maternal pyrexia or chorioamnionitis, date of delivery, gestational age at delivery and all neonatal data including birthweight, length of stay at NICU and morbidity related to prematurity.

The following methodological data will also be requested for all trials: method of generation of randomisation list, method of concealment of randomisation, stratification factors and blinding methods.

Data will be accepted either in electronic (floppy disk/CD/internet) or paper form. A desired format and coding will be specified but trialists may supply data in the most convenient way open to them, providing details of coding are sent with the data.

### Data validation strategy

A copy of the original data sent (before checking) will be held in a separate file. The following procedures will then be performed and documented for all trial data supplied. Trial details will be crosschecked against any published report of the trial. Range and consistency checks will be applied – missing data, errors and inconsistencies will be followed up with a nominated individual. The chronological randomisation sequence will be reviewed. The balance of prognostic factors will be checked, taking into account of factors stratified for in the randomisation procedure.

### Outcome measures

The primary outcome of interest will be neonatal mortality/morbidity. Choice of primary outcome is about what should determine clinical decision-making. However it is recognised that trials to date may have insufficient power and there is a need to consider secondary outcomes of time from randomisation to delivery, preterm delivery before 32 completed weeks (<32+0 weeks) and maternal morbidity as defined in the Cochrane Review (Drakeley 2003)[1]. We will aim to obtain all neonatal and maternal morbidity outcome data collected in each trial and not just those reported in publications.

Reporting of these outcomes in the original trial report is not an eligibility requirement for this review.

### Data analysis

Data on all randomised patients will be requested to perform an intention-to-treat analysis as far as possible. Clinical heterogeneity will be assessed by reviewing the differences across trials in characteristics of randomised patients.

Initially, an aggregate data analysis will be undertaken although treatment effect estimates will be obtained from the individual patient data. Binary outcomes will be summarised in terms of odds ratios or relative risks, depending on the degree of heterogeneity observed. Time-to-event outcomes will be summarised in terms of the log (hazard ratio). The I square statistic and chi-square test for statistical heterogeneity will be applied to these summary data.

Regression models, stratified by trial, will be used to explore the effects of treatment, risk factors and treatment-covariate interactions on the various outcomes of interest. These will include Cox and accelerated life models for time-to-event outcomes (Tudur-Smith 2004 [18], Williamson 2002 [19]) and logistic regression models with trial indicator variables for binary outcomes (Whitehead 2002) [20]. Factors other than treatment to be investigated are gestational age at randomisation, maternal demographics, obstetric characteristics including obstetric history, cervical length on ultrasound, fibronectin, bacterial vaginosis, multiple pregnancy.

Two-level multilevel regression models will be fitted with patients corresponding to level one units and trials as level two units for the various outcomes of interest, adopting the relevant approach for continuous, binary, categorical and time to event outcomes as applicable. Trial effects will be represented by fixed effects whilst treatment effects will be represented by random effects in an attempt to reflect the assumed similar (but not identical) treatment effect across trials. Patient-level covariates (as listed above) will then be incorporated into the model in an attempt to account for some of the remaining statistical heterogeneity. An attempt will be made to incorporate these covariates first of all by assuming their effect to be constant across all trials and subsequently by assuming some heterogeneity in the covariate effect across trials by modelling them either as fixed or random effects. Finally, treatment-covariate interactions will be investigated by including additional variables and adopting a similar approach.

If IPD are not available for some trials, the potential for bias will be investigated as follows. The reasons for not being able to obtain the data will be assessed for the potential for bias. Results using aggregate data from these trials will be compared with results using aggregate data from trials where IPD have been supplied, and any difference investigated. The analysis plan will be reviewed in light of the availability of IPD but prior to any comparative analyses.

## Discussion

Predictive models generated by our analysis should allow more effective counselling of women at risk of preterm delivery and thus more cost effective use of cerclage.

## Competing interests

ZA and PRW were authors of a paper that will be included in the IPD meta-analysis (To, 2004). ZA was an author of the non-IPD systematic review on this topic (Drakeley, 2003)[1]. The authors declare that they do not have any other competing interests.

## Authors' contributions

PRW conceived the idea, CTS drafted the initial protocol, and all authors commented on and approved this final version.

## Pre-publication history

The pre-publication history for this paper can be accessed here:


